# The impact of patient feedback on the medical performance of qualified doctors: a systematic review

**DOI:** 10.1186/s12909-018-1277-0

**Published:** 2018-07-31

**Authors:** Rebecca Baines, Sam Regan de Bere, Sebastian Stevens, Jamie Read, Martin Marshall, Mirza Lalani, Marie Bryce, Julian Archer

**Affiliations:** 1 Collaboration for the Advancement of Medical Education Research & Assessment (CAMERA), Faculty of Medicine and Dentistry, University of Plymouth, Drake Circus, Plymouth, PL4 8AA UK; 2 Improvement Science London, University College London, London, UK; 3 Research Department of Primary Care and Population Health, University College London, London, UK

**Keywords:** Patient feedback, Systematic review, Medical education, Impact, Behaviour change, Doctors

## Abstract

**Background:**

Patient feedback is considered integral to quality improvement and professional development. However, while popular across the educational continuum, evidence to support its efficacy in facilitating positive behaviour change in a postgraduate setting remains unclear. This review therefore aims to explore the evidence that supports, or refutes, the impact of patient feedback on the medical performance of qualified doctors.

**Methods:**

Electronic databases PubMed, EMBASE, Medline and PsycINFO were systematically searched for studies assessing the impact of patient feedback on medical performance published in the English language between 2006-2016. Impact was defined as a measured change in behaviour using Barr’s (2000) adaptation of Kirkpatrick’s four level evaluation model. Papers were quality appraised, thematically analysed and synthesised using a narrative approach.

**Results:**

From 1,269 initial studies, 20 articles were included (qualitative (*n*=8); observational (*n*=6); systematic review (*n*=3); mixed methodology (*n*=1); randomised control trial (*n*=1); and longitudinal (*n*=1) design). One article identified change at an organisational level (Kirkpatrick level 4); six reported a measured change in behaviour (Kirkpatrick level 3b); 12 identified self-reported change or intention to change (Kirkpatrick level 3a), and one identified knowledge or skill acquisition (Kirkpatrick level 2). No study identified a change at the highest level, an improvement in the health and wellbeing of patients. The main factors found to influence the impact of patient feedback were: specificity; perceived credibility; congruence with physician self-perceptions and performance expectations; presence of facilitation and reflection; and inclusion of narrative comments. The quality of feedback facilitation and local professional cultures also appeared integral to positive behaviour change.

**Conclusion:**

Patient feedback can have an impact on medical performance. However, actionable change is influenced by several contextual factors and cannot simply be guaranteed. Patient feedback is likely to be more influential if it is specific, collected through credible methods and contains narrative information. Data obtained should be fed back in a way that facilitates reflective discussion and encourages the formulation of actionable behaviour change. A supportive cultural understanding of patient feedback and its intended purpose is also essential for its effective use.

**Electronic supplementary material:**

The online version of this article (10.1186/s12909-018-1277-0) contains supplementary material, which is available to authorized users.

## Background

Patient feedback is considered integral to quality improvement and professional development [1–3]. Designed to guide behaviour change and facilitate reflective practice [4], patient feedback is increasingly incorporated into medical education including continuing professional development and regulatory initiatives such as medical revalidation [5–9]. Typically collected as part of a questionnaire based assessment [10, 11], patient feedback tools have been validated across a range of specialities and geographical locations including Canada, the USA, Netherlands and Denmark [10]. However, their inclusion in regulatory initiatives and other educational activities is not without its criticisms, with current literature to support its impact on medical performance particularly limited in a post-graduate setting [11, 12].

Kluger and others critique the importance placed on patient feedback as a performance assessment methodology due to the implicit and often unclear assumptions made about its capacity to facilitate quality improvement [11, 13–15]. The quality of evidence used to support its capacity to facilitate change is also frequently called into question [13]. As Lockyer et al. notes, notwithstanding the considerable amount of research examining the psychometric properties of specific patient feedback tools, current understanding of patient feedback as a catalyst for change remains limited [12]. Little attention has been paid to the formative or educational impact of patient feedback on doctor performance [10, 11, 16, 17]. As Ferguson and others note, further research is needed to establish if, and how, patient feedback influences doctor i.e. physician or resident behaviour and to identify which factors may have greatest influence [13].

As a result, in line with international efforts to incorporate patient feedback into regulatory and other educational initiatives [7, 18], we undertook a systematic review to: i) assess if, and how, patient feedback is used by the medical profession; ii) identify factors influential in determining its efficacy and; iii) identify any potential challenges or facilitators surrounding its impact on medical performance. Our review specifically sought to address the following research questions: what impact does patient feedback have on the medical performance of individual doctors, and what factors influence its acceptance in a medical environment?

For this review, we use the term ‘patient’ to be inclusive of service-users, consumers, carers and/or family members although the important distinctions between these terms is acknowledged. We define patient feedback as information provided about an individual doctor through formal patient experience or satisfaction surveys/questionnaires e.g. multi-source feedback (MSF) or patient feedback assessments but exclusive of formal complaints, online platforms or feedback beyond the service of an individual doctor i.e. healthcare team or service.

## Methods

To ensure transparency of findings, our review followed the Preferred Reporting Items for Systematic Reviews and Meta-Analysis (PRISMA) [19], and Centre for Reviews and Dissemination guidance [20].

### Search Strategy

Using the SPICE framework [21], one research team member (RB) designed the search terms listed in Table 1. All search terms were reviewed by the wider team in line with the Peer Review of Electronic Search Strategies (PRESS) guidance to maximise sensitivity and specificity [22]. As advised by an information specialist, we searched Medline, EMBASE, PsycINFO and PubMed databases for articles published in the English Language between January 2006 and December 2016. This date parameter was selected to ensure the most contemporary information was included. Electronic searches were supplemented with citation searches and reviewing reference lists of eligible studies. Duplicate studies were removed electronically and double checked by another research team member (SS). Two independent reviewers conducted the research process.

**Table 1:** Systematic review search strategy

Search strategy	
**Setting:** “physician” OR “doctor” OR “surgeon” AND **Perspective**: “doctor” OR “physician” OR “surgeon” OR “patient*” OR “user” OR “client” OR “consumer*” OR “survivor” OR “representative*” OR “family” OR “relative” AND **Intervention**: “multisource feedback” OR “multi-source feedback” OR “360 degree feedback” OR “360 degree evaluation” OR “MSF” OR “performance feedback” OR “PF” OR “patient experience” OR “patient survey” OR “patient questionnaire” AND **Evaluation**: “professional development” OR “behaviour change” OR “improve” OR “quality of care” OR “learn*” OR “reflect” OR “impact” OR “outcome” OR “patient safety”	

### Study selection

We selected studies through a two-stage process. Firstly, two reviewers (RB, SS) independently examined titles and abstracts using Rayyan, a web application for systematic reviews [23]. To ensure inclusion/exclusion standardisation, reviewers used a piloted inclusion criteria form [Additional file 1]. When a selection decision could not be made, the full article was retrieved. Potentially relevant articles were then independently assessed by two researchers (RB, SS). If any discrepancies arose these would have been resolved by discussion with a third reviewer (JR) until consensus was achieved. This process was not required during the research process.

### Inclusion/exclusion criteria

Studies published in the English language between 2006-2016, exploring the impact of patient feedback on medical performance in any healthcare setting using any study design except opinion, commentary or letter articles were included. Due to resource constraints studies published in languages other than English were excluded, as were those outside the pre-defined date parameters to ensure only the most contemporary evidence was reviewed. Studies that solely discussed the psychometric properties of specific patient feedback tools were excluded due to the review focusing on reported change in medical performance.

Where studies discussed the impact of MSF or work-placed based assessment more broadly but included findings about patient feedback which could be clearly identified, these were included. If it was not possible to differentiate the specific influence of patient feedback from other feedback sources, the article was excluded to avoid result dilution. Finally, due to our area of interest, studies in the context of undergraduate medical education and methods of patient feedback not currently accepted in regulatory processes such as online feedback sites were excluded.

### Data extraction and outcomes

Two reviewers (RB, SS) independently undertook data extraction of all included studies using a piloted data extraction form. Information extracted included: year published; study location, aim design, population, and methodology. In order to address our research questions, we used Barr’s (2000) adaptation of Kirkpatrick’s four level evaluation model [Additional file 2] to evaluate study outcomes [24]. Where studies covered MSF or work-place based assessments more broadly, only those findings relating specifically to patient feedback were extracted for review inclusion.

### Quality assessment

Two research team members (RB, SS) independently assessed study quality using: the Critical Appraisal Skills Programme Qualitative checklist [25]; Quality Assessment instrument for observational cohort and cross-sectional studies [26]; and Quality Assessment of Systematic reviews and Meta-analyses [27]. Due to the focus of this review not relying solely on the methodological quality of included studies, conceptual relevance took precedence over methodological rigour [28]. However, we conducted sensitivity analyses to assess the impact of study quality on review findings [29, 30]. Sensitivity analyses test for the effect of study inclusion/exclusion on review findings [29]. It is considered an important focus of any review synthesis involving qualitative research [31].

### Data analysis and synthesis

Data were analysed using an inductive thematic analysis approach [29, 32]. The team initially reviewed two papers to develop a comprehensive coding framework. The framework was then used to individually analyse all included studies and to iteratively compare emerging themes across studies to determine dominant themes. We then synthesised themes using a modified narrative synthesis technique grounded in Popay et al’s. guidance [33].

## Results

**Fig. 1:**
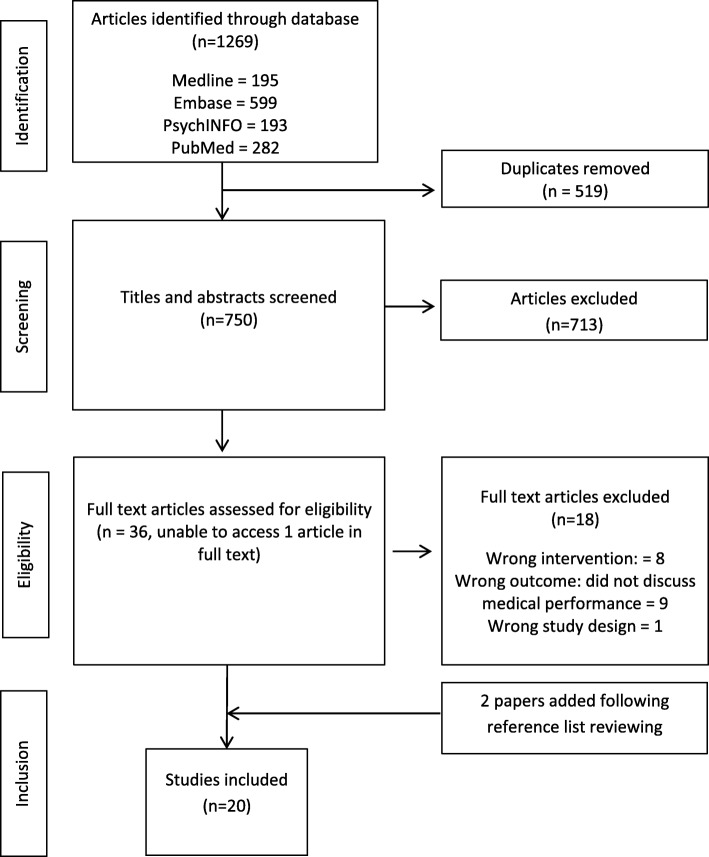
PRISMA flow diagram

From an initial identification of 1,269 articles, 36 studies were considered potentially relevant. Of these, 18 were excluded due to irrelevant: study design [34]; intervention; [6, 35–42] or outcome i.e. did not discuss medical performance impact. [5, 16, 43–49] A total of 18 articles supplemented with two articles found through reference list searching were included for the purposes of this review (Fig. 1). Results are discussed in order of study characteristics; study quality; impacts of patient feedback on medical performance; and factors found to influence the use of patient feedback to improve medical performance.

### Study characteristics

We included studies with a variety of study designs including: qualitative methodologies (*n*=8); observational designs (*n*=6); systematic reviews (*n*=3); mixed methodologies (*n*=1); one randomised control trial; and a longitudinal study. Studies primarily focused on family doctors (*n*=6). Other populations studied included: unspecified doctor groups or settings (*n*=5); residents (*n*=3); consultants (*n*=2); medical specialists (*n*=1); primary care groups leaders (*n*=1), doctors (*n*=1); and department chiefs (*n*=1). Studies were conducted in: Canada (*n*=6); the UK (*n*=5); US (*n*=4); Netherlands (*n*=4) and Denmark (*n*=1). A summary of study characteristics is provided in Table 2.

**Table 2:** Included study characteristics

Author	Publication date	Study Location	Study Design	Study population and sample methodology	Intervention type	Feedback delivery	Facilitated feedback	Barr’s Kirkpatrick level of change
Friedberg et al., [7]	2010	USA	Qualitative: semi-structured interviews	Volunteer sample of 72 adult primary care group leaders including medical directors, administrators, or managers in a hospital setting	Patient experience Survey: Massachusetts Health Quality Partners	“Detailed report”	No	4a
Brinkman et al [50].,	2007	USA	RCT	Volunteer sample of 36 first year paediatric residents in a hospital setting	MSF	Report including narrative comments and comparison data	Yes	3b
Rodriguez et al. [51],	2009	USA	Observational	Volunteer sample of 145,522 randomly selected patients with encounters of 1,444 primary care physicians	Patient survey	Report	Not specified	3b
Fustino et al. [52],	2015	USA	Observational	Opportunity sample of four full-time physicians working in an outpatient setting	Patient feedback intervention following Press-Ganey patient satisfaction survey	Weekly email reports	Yes	3b
Violato et al., [53]	2008	Canada	Longitudinal	Convenience sample of 250 (169 men, 81 women) randomly selected GPs, or family doctors.	MSF:PAR	Report	Yes	3b
Ferguson et al. [13],	2014	UK	Systematic review	N/A	MSF	Report including aggregate scores and comparison data	Mixed	3a-3b
Reinders et al. [54],	2011	Netherlands	Systematic review	N/A	Patient feedback	Report	Not specified	1-3b
Overeem et al. [55],	2012	Netherlands	Observational	Volunteer sample of 236 (144 men, 92 women) medical specialists	360-degree feedback	Report	Yes: mentor	3a
Overeem et al. [10],	2010	Netherlands	Observational	Volunteer sample of 109 consultants working in a hospital setting	MSF: PAR, ABIM, AAI, completion of a descriptive and reflective portfolio, assessment interview and personal development plan.	Report	Yes: “trained facilitator”	3a
Nielsen et al. [56],	2013	Denmark	Observational	Volunteer sample of 32,809 inpatients survey responses. Volunteer sample of department heads, and purposeful sampling of 24 representatives (eight doctors, eleven nurses, and five managers)	Patient surveys	Report	Not specified	3a
Lewkonia et al. [8],	2013	Canada	Observational	Purposeful volunteer sample of 51 family physicians and general practitioners	MSF: PAR and facilitated practice visit	Report	Yes	3a
Overeem et al. [57],	2009	Netherlands	Qualitative: semi-structured interviews	Purposeful sampling of 23 (14 male, 9 female) consultants working in a hospital setting	MSF: PAR and ABIM, portfolio construction and personal development plan including improvement goals	Report with narrative comments	Yes: trained facilitator	3a
Sargeant et al. [58],	2011	UK	Qualitative: semi-structured interviews	Volunteer sample of 13 GP trainer and trainees	MSF	Report	Yes: trainers	3a
Sargeant et al. [59],	2009	Canada	Qualitative: semi-structured interviews	Volunteer sample of 28 purposefully sampled family physicians	MSF: NSPAR	Mailed report	No but could be requested	3a
Sargeant et al. [2],	2008	Canada	Qualitative: semi-structured interviews	Volunteer sampling of 28 (22 men, 6 women) purposefully sampled family physicians	MSF:PAR	Report	No but requested	3a
Sargeant et al. [60],	2007	Canada	Qualitative: semi-structured interviews	Volunteer sample of 28 doctors (12 high performing, 16 in the average /lower-scoring group) identified through purposeful sample	MSF: PAR	Mailed reports	No	3a
Edwards et al. [11],	2011	UK	Qualitative: semi-structured interviews	Stratified volunteer sample of 30 general practitioners (21 males and 9 females)	Patient-experience survey: including CPAQ and IPQ	Report	Not specified	3a
Miller et al. [17],	2010	UK	Systematic review	N/A	Workplace based assessment	Report	Not specified	3a
Wentlandt et al. [61],	2015	Canada	Mixed methods	Volunteer sample of 4 department chiefs and 12 physician participants (9 men, 3 women)	The Physician Quality Improvement Initiative (PQII)	Report	Yes: department chief	3a
Burford et al. [1],	2011	UK	Qualitative: telephone interviews	Volunteer sample of 35 junior doctors (13 male, 22 female [6 F1 doctors, 29 F2]), and random sample of 40 GP patients (20 male, 20 female)	Patient experience survey: Doctors interpersonal skills questionnaire (DISQ)	Report	Yes, if sent directly to supervisor	2b

### Study quality

We found the methodological quality of included studies to be mixed. Studies were considered high (*n*=13), acceptable (*n*=6), and low (*n*=1). Although low, sensitivity analysis identified that its inclusion had no impact on the review synthesis and did not therefore dilute conclusions drawn.

### Impact of patient feedback on medical performance

Included studies reported: a change in organisational practice (*n*=1, Kirkpatrick level 4a) [7]; a measured change in behaviour (*n*=6, Kirkpatrick level 3b) [13, 50–54]; self-reported change or intention to change (*n*=12, Kirkpatrick level 3a), [2, 8, 10, 11, 17, 55–61] and acquisition of knowledge or skills (*n*=1, Kirkpatrick level 2b) following the provision of patient feedback [1]. No studies identified a change at the highest evaluation level – a change in the health and wellbeing of patients (Kirkpatrick level 4b). These are discussed in turn below.

#### Measured change

We found mixed results for the evidence of measured change in medical performance. For example, an RCT demonstrated an increase in patient ratings for both the control (*n*=18), and intervention group (*n*=18) on items relating to communication and shared decisions [50]. Participants in the intervention group (who participated in tailored coaching sessions) also experienced statistically significant increases in four additional items: being friendly; being respectful; showing interest; and explaining problems. However, in contrast to nurse ratings, the overall difference in patient ratings between groups did not reach statistical significance [50]. It is unclear which element of the MSF intervention e.g. the MSF itself or tailored coaching led to the measured changes, preventing an assessment of potential causation. A longitudinal study investigating changes in medical performance as assessed by patients, co-workers and medical colleagues also identified significant changes (small-moderate) in colleague and co-worker assessments, but not that of patient feedback [53].

Conversely, other studies reported significant improvements in patient feedback [51, 52, 54]. One observational study assessing the impact of financial incentives on patient experience for individual doctors identified significant improvements in: doctor-patient communication [95% confidence interval (CI): 0.61, 0.87, *p*<0.001]; care coordination (0.48; 95% CI: 0.26, 0.69); and office staff interaction (annual point change=0.22; 95% CI: 0.04, 0.40, *p*=0.02) over a period of three years [51]. Doctors with lower baseline performance scores typically experienced greater improvements (*p*<0.001). Similarly, incentives that placed greater emphasis on clinical quality and patient experience were associated with larger improvements in care coordination (*p*<0.01) and office staff interaction (*p*<0.01). In contrast, incentives emphasising productivity and efficiency were associated with declines in doctor communication performance (*p*<0.01) and office staff interaction (*p*<0.01) [51].

#### Self-reported change

Similar to the results of measured change studies, self-report studies appear mixed in terms of patient feedback use and efficiency and typically identify a small-moderate change. [2, 8, 55, 58, 60] In one study, 78% (40/51) of primary care doctors reported making a practice change following patient feedback results [8], but most included studies reported a smaller effect [55, 60]. For example, in one study where participants received average-lower scores (13/28), 54% (7/13) reported making a change [60]. However, 54% (15/28) of participants from the same study also reported making no change; [60] highlighting the variability of patient feedback impact [2, 13, 17, 57, 59, 60]. Some included studies reported no intention to change [10, 57, 61].

#### Change in knowledge/skill acquisition

One study identified a change in knowledge acquisition/understanding [1]. Doctors involved in this study reported learning about the importance of trust, consultation style and communication [1].

#### Improvements or changes made

Finally, while all 20 studies reported a change in medical performance to some degree, 13 identified specific changes in behaviour. Communication was the most frequently targeted area for improvement [1, 7, 13, 50–54, 60, 61]. Few identified initiatives targeting clinical competence, care coordination [51], or access to healthcare services [7, 52].

#### Factors found to influence the use of patient feedback to improve medical performance

Several studies identify the source, content and delivery of patient feedback to be influential in its assimilation, acceptance and use. Specifically, its: perceived credibility; congruency with self-perceptions and performance expectations; presence of facilitation and reflective discussions; and inclusion of narrative comments.

#### Feedback source

Nine studies reviewed described the perceived credibility of patient feedback as influential [2, 8, 10, 11, 13, 17, 56, 58, 60], particularly when feedback was considered negative in nature [2, 11, 13]. Doctors who received negative feedback typically placed greater emphasis on the assessment process; often citing such factors as reasons behind non-acceptance [2]. Similar findings are also reported in Ferguson et al.’s review where doctors questioned feedback credibility and postponed behavioural change until the process had been verified by conducting their own independent reviews [13].

Feedback is also more likely to be incorporated into behaviour change efforts when a doctor considers the rater to be familiar and able to observe their practice [13, 17, 60]. Sargeant et al. reported that doctors who made a change did so in response to patient feedback preferentially over that of medical colleagues [60]. Conversely, research conducted by Edwards et al., identified ambiguity surrounding the credibility of patient feedback [11]. Doctors interviewed highlighted concerns that patients were completing feedback surveys on the basis of their general views of the doctor as a person and not that of their medical performance [11]. Similarly, Overeem reported that only the mean ratings of colleagues (r=-0.195, *p*<0.01) and self-ratings (r=-0.179, *p*<0.05) and not those of patients were significantly correlated with reported behaviour change [10].

#### Feedback content

Factors identified as influential in terms of feedback content included: feedback specificity; a perceived need for change; and consistency with other feedback sources [2, 11, 13, 53, 60, 61]. However, we found that the most influential factor identified by eight included studies was feedback congruency between a doctors’ self-perception and performance expectation [2, 10, 11, 13, 55, 58–60]. As described by Sargeant et al., feedback interpreted as positive is typically congruent with ones’ self-perception or expectations whereas, feedback interpreted as negative is typically incongruent with such perceptions [59]. Both forms of feedback may be troublesome to incorporate into behaviour change [11, 58–60]. Edwards et al., reported that feedback considered above average, i.e. positive, rarely led to actionable change as it was simply considered a positive affirmation of practice [11]. Conversely, negative feedback, tends to elicit greater emotional reactions and extended periods of reflection, that may, or may not, led to eventual acceptance [59]. For example, doctors interviewed two years after receiving feedback inconsistent with self-perceptions reported the same emotional and reflective reactions as experienced two years before [2].

#### Feedback delivery: facilitation and reflection

Early access to facilitated reflective discussions that explore emotional reactions appear integral to feedback assimilation, acceptance and subsequent use [2, 10, 11, 13, 17, 58, 59, 61, 62]. Several studies described how facilitation can support feedback acceptance and encourage achievable goal setting [2, 10, 13]. Studies that failed to provide facilitated feedback indicated a need for such an activity [13]. In one instance, a series of recorded discussions between trainees and trainers about a MSF report found trainers used open-ended questions to initiate reflective discussions and subsequent behaviour change initiatives [58]. Such openness and encouragement was widely appreciated by interviewed trainees and accepted as a way to enable unanticipated learning [58]. Identified benefits specifically related to facilitated reflective discussions include: reduced anxiety; more timely processing of patient feedback; validation of emotional reactions; prevention of jumping to premature or potentially incorrect conclusions; and increased ability to identify specific change needs [58, 59, 61].

#### Facilitation quality

However, perceived mentor quality can limit the facilitation of patient feedback [58, 61]. Research conducted by Overeem et al., suggests consultants who identified specific facilitator skills including reflection, encouragement and specificity in goal setting were key to behavioural change [10]. Consultants who attained higher levels of improvement regularly identified these facilitator skills [10].

#### Narrative comments

The inclusion of narrative comments was influential in supporting behaviour change [10, 13, 58]. Evidence reviewed suggests participants prefer to receive written comments as opposed to numerical scores only, and that there is a small, yet significant, preference for free text comments, with written comments from raters considered essential to physician satisfaction and patient feedback use [13]. Furthermore, an analysis of interview transcripts discussing MSF reports by Sargeant et al., reveals that trainers and trainees do not typically discuss the numerical scores, but focus their discussion predominately on the narrative comments provided [58].

#### Medical culture

The existing medical culture may complicate behaviour change efforts [2, 10, 56, 57]. As acknowledged by Nielsen et al., norms that originate within the medical community, including a lack of openness and social support, may restrict performance initiatives [56]. Sargeant et al. described how many doctors interviewed discussed the influential nature of the professional culture on performance expectation and subsequent feedback acceptance [60]. Participants spoke of “being a doctor” and how this identity made it particularly important to be viewed positively by others. The authors explain how the collective, and individual desire for doctors to “do good,” leads to doctors holding a high expectation of providing above average care [60]. Feedback that challenges this self-perception is then often difficult to assimilate. Furthermore, while self-directed practice is considered the norm in medicine, being assessed in practice is typically not [2].

Finally, Nielsen argues that hospital environments and other medical settings leave little room for rational patient-centred change, due to competition with other more clearly specified institutional norms [56]. Overeem reports that consultants are not strongly motivated to use feedback to improve medical performance as they see feedback exercises as a means to enhance public trust, and not one to incentivise performance improvement [10, 57]. Overeem concludes that one of the most frequently experienced barriers to behavioural change is working in an environment unconducive to lifelong reflective learning [10, 57].

## Discussion

Our review responds to calls for further research to establish if, and how, patient feedback impacts on medical performance and to identify factors influential in this process [63]. While several existing reviews have explored the impact of workplace based assessments and MSF more broadly, to date, no reviews have focussed specifically on the educational impact of patient feedback beyond consultation or communication skills. Our review findings suggest patient feedback has the potential to improve medical performance, but the level at which behaviour change occurs as assessed by Kirkpatrick’s evaluation model varies. No included study identified a change at the highest evaluation level, a change in the health and wellbeing of patients. Longer term studies that explore the relationship between patient feedback and impact are needed, as is the examination on patient wellbeing, although the difficulties of achieving this are acknowledged [17, 54].

Our proposed explanation for the behavioural change variability reported is the presence, or absence, of factors identified as influential in patient feedback acceptance, use and assimilation. Specifically, its: perceived credibility; specificity; congruency; presence of facilitated and reflective discussion; and inclusion of narrative comments. Patient feedback is more likely to initiate behaviour change if participants: consider the process, instrument and provider to be credible; receive feedback that is consistent with self-perceptions or performance expectations; are able to identify specific behavioural change measures through reflective discussions; discuss their feedback with a skilled facilitator who use open ended questions to facilitate reflective discussions and behaviour change and receive narrative comments.

The value of narrative feedback is acknowledged across postgraduate and undergraduate settings due to the unadulterated information they provide over and above that provided in numerical scores or grades [64–66]. Although not without its difficulties, [67, 68] there is increasing evidence to suggest recipients can interpret comments and use them to modify their performance [69, 70]. Recent research also highlights the “stark contrast between survey scores and comments provided” [64], with patients often awarding highly positive or inflated scores [66], in addition to conflicting negative narrative comments. A focus on inflated scores could mislead professional development efforts and diminish the apparent need for continued improvement. Opportunities for reflective learning and professional development may therefore lie in narrative feedback as opposed to numerical scores, an element existing feedback tools currently rely on with limited scope or room for narrative feedback inclusion. Similar to Sargeant et al.’s research in a trainee setting, future research should examine the content and focus of feedback discussions when reviewing patient feedback reports. Is there an equal discussion between the numerical scores and narrative comments, or does one domain take precedence over the other? Based on the evidence reviewed, narrative feedback should be incorporated into current and future feedback tools across the education continuum to encourage reflective practice and beneficial behaviour change where required.

As part of the contextual landscape in which patient feedback is received, we found that facilitated reflection appears integral to transforming initial patient feedback reactions into measurable behavioural change, quality improvement initiatives or educational tasks [11, 58]. With this in mind, receiving feedback in isolation of reflective and facilitated discussions may not be enough to bring about immediate or sustained change to the betterment of professional development and subsequent patient care [71]. This alongside the highlighted importance of facilitator quality has important implications for the recent Pearson review into medical revalidation in the UK where the importance of reflective discussions was identified; “it’s [feedback] only useful if the quality of the appraiser/appraisal is good and there is appropriate reflection at appraisal.” [18] Facilitated discussions where reflection is supportively encouraged appears integral to dealing with emotional responses and transforming initial reactions into measurable behavioural change.

Finally, one factor that appears relatively unexplored in the existing literature is the influence of cultural context [71]. Encouraging a culture that promotes constructive feedback and reflection-in-action could enable performance improvement more readily [10, 58]. As reported by Pearson, medical revalidation is currently “at the acceptance stage, and the next step is to strengthen ownership by the profession, and engagement with the public” ^P.38^ [18]. Wider engagement of patients and the public as suggested may provide the cultural change catalyst needed to support behavioural change and educational outcomes. However, it is notable that we did not find any literature on patient feedback from a patient perspective. Assumptions are often made about the desire of patients and the public to feedback on their doctors i.e. on what, how and when, but exploration of these issues have been little explored. Organisations and institutions that use patient feedback as a form of performance evaluation should seek to alter existing cultures, enabling the collection of patient feedback to become a valued and embedded activity. This will need to include an honest and protected space in which to allow doctors to openly reflect and where needed, acknowledge error without fear and consequence [72, 73].

Strengths of this review include its application of a recognised systematic review process, [19, 20] and utilisation of Kirkpatrick’s evaluation model to provide greater insight into the impact of patient feedback. However, its limitations must also be acknowledged. The methodological quality of some included studies is somewhat undermined by the voluntary nature, and in some cases, small sample size of participant populations. Acknowledged limitations of this sampling method include potentially biased or highly motivated participants whose results may not generalise to the wider population. Most studies are also non-comparative or observational. The conclusions drawn may therefore be limited by their uncontrolled nature. However, assessing behavioural or educational impact on the medical performance of individual doctors is difficult to achieve [54]. For example, few studies differentiate between medical practice and educational improvements, or clearly define these parameters. Furthermore, descriptive or observational studies provide useful information in the exploration of complex interactions therefore warranting their inclusion [17]. The predominance of qualitative or observational methodologies in this review should not therefore be seen as a significant limitation. Despite its frequent use in medical education, Kirkpatrick’s framework is also not without its critics [74, 75]. Furthermore, three studies by Sargeant et al. [2, 59, 60], draw on the same sample population leading to possible publication bias. Some systematic reviews included in this article also report on the same primary studies, leading to possible result duplication. Finally, although an extensive review of published literature was undertaken, grey literature was not included and relevant non-peer reviewed studies may therefore not be included.

Future research should explore the feasibility of conducting a realist review [76] to help further unpick the complexity of patient feedback and to identify what works for whom, and in what circumstances. Realist reviews are increasingly being adopted in other areas of medical education including doctor appraisal [77] and internet based education [78].. To the authors’ knowledge, a realist review of patient feedback in medical education has yet to be completed highlighting a gap in existing knowledge.

## Conclusion

This review holds import implications for the use of patient feedback across the educational continuum. Patient feedback can have an impact on medical performance. However, its acceptance, assimilation, and resultant change, are influenced by a multitude of contextual factors. To strengthen patient feedback as an educational tool, initiatives should be: specific; collected through credible methods; contain narrative comments; and involve facilitated reflective discussions where initial emotional reactions are processed into specific behavioural change, quality improvement initiatives or educational tasks. Understanding and encouraging cultural contexts that support patient feedback as an integral component of quality improvement and professional development is essential. Future patient feedback assessment tools should be accompanied by facilitated discussion that is of high quality.

## Additional files

Additional file 1. Piloted inclusion form. (DOCX 16 kb)

Additional file 2. Barr's (2000) adaptation of Kirkpatrick's four level evaluation model. (DOCX 15 kb)
