# Supplementary material for: The impact of patient feedback on the medical performance of qualified doctors: a systematic review
Source: BMC Med Educ. 2018 Jul 31;18:173. doi: 10.1186/s12909-018-1277-0 (PMC6069829; doi:10.1186/s12909-018-1277-0)
Supplement: Supplementary file 1 — Piloted inclusion form. (DOCX 16 kb) [file 12909_2018_1277_MOESM1_ESM.docx]

Additional file 1: piloted inclusion form

| **Inclusion criteria form** |
| --- |
| 1. Is the study available in English?   Yes (proceed) No (reject)   1. Is the study published between 2006 and 2016?   Yes (proceed) No (reject)   1. Does the study talk about the impact of patient feedback on medical performance?   Yes (proceed) No (reject)   1. Is the study related to online/social media comments?   Yes (reject) No (proceed)   1. Is the study solely focused on the psychometric properties of a patient feedback tool?   Yes (reject) No (proceed)   1. Is the study focused on patient feedback in the context of undergraduate medical education?   Yes (reject) No(proceed) |
