# Supplementary material for: The impact of patient feedback on the medical performance of qualified doctors: a systematic review
Source: BMC Med Educ. 2018 Jul 31;18:173. doi: 10.1186/s12909-018-1277-0 (PMC6069829; doi:10.1186/s12909-018-1277-0)
Supplement: Supplementary file 2 — Barr's (2000) adaptation of Kirkpatrick's four level evaluation model. (DOCX 15 kb) [file 12909_2018_1277_MOESM2_ESM.docx]

Additional file 2: Barr's (2000) adaptation of Kirkpatrick's four level evaluation model

| Level | Description |
| --- | --- |
| **Level 1: learners’ reaction** | Relate to participants views of their learning experience programme |
| **Level 2: learning outcomes** |  |
| 2a: modification of attitudes/perception | Changes in reciprocal attitudes or perceptions between participant groups, towards patients/clients and their condition, circumstances, care and treatment. |
| 2b: Acquisition of knowledge/skills | Acquisition of concepts, procedures and principles of inter-professional collaboration or the acquisition of thinking/problem-solving, psychomotor and social skills linked to collaboration |
| **Level 3: Change in behaviour**  3a: Self-reported change in behaviour Level  3b: Measured change in performance | Behavioural change transferred from the learning environment to the workplace prompted by modifications in attitudes or perceptions, or the application of newly acquired knowledge/skills in practice. Overeem et al. (2010) identify that this level can be further separated into: |
| **Level 4: Patient/Organisational outcomes** |  |
| 4a: Change in organisational practice  4b: Benefits to patients/clients | This relates to wider changes in the organisation/delivery of care, attributable to an education programme.  Covers any improvements in the health and well-being of patients/clients as a direct result of an education programme. |
